# Dynamic feature selectivity in the thalamus of the rat whisker system

**DOI:** 10.1186/1471-2202-14-S1-P372

**Published:** 2013-07-08

**Authors:** Clarissa J Shephard, Daniel C Millard, Garrett B Stanley

**Affiliations:** 1 Wallace H Coulter Department of Biomedical Engineering, Georgia Tech and Emory, Atlanta, GA 30336, USA

## 

As information progresses along the sensory pathway, it is transformed from physical attributes of a stimulus at the periphery to complex representations at the level of cortex that ultimately give rise to perception. Neurons at each stage in the sensory pathway are commonly characterized by simple linear models that describe the physical attribute of the stimulus that maximally excites the cell. However, neurons, even early stages of processing, can have strongly nonlinear characteristics that give sensory systems the remarkable capability to extract relevant information (features) from noisy environments (context conditions). In this work, we explored the bottom-up effect of stimulus context on the encoding of features in the whisker thalamocortical circuit of the anesthetized rat. The feature selectivity of thalamic neurons was quantified using spike triggered analysis techniques under three different stimulus context conditions (low, medium, and high amplitude Gaussian distributed white noise mechanical stimulation). After characterizing each cell, the response to three features (position, velocity, and acceleration) embedded in each context condition were recorded, and ROC analysis was utilized to quantify the detectability of the stimulus in the presence of increasing levels of background noise. Neural responses to features embedded in each context condition were then analyzed as a function of the similarity between the embedded feature and the estimated feature selectivity of the cell to assess the discriminability of the feature from the background. Preliminary findings suggest that while the estimated feature selectivity is generally predictive of the thalamic response to presentation of isolated features, the linear models alone are not predictive of the thalamic response to features in different noise contexts. We hypothesize that this is due to a recruitment of nonlinear circuit properties by the higher amplitude noise stimuli. This hypothesis is supported by the reduction of spike timing reliability in the thalamic neurons as the noise amplitude increases, a finding that mirrors results in the visual thalamus and has been attributed to nonlinear circuit mechanisms [1]. Future work is aimed at elucidating the effect of stimulus context on feature encoding in varying signal-to-noise ratio conditions in the thalamocortical circuit and ultimately the effect on perception.
